# The Pyrazolo[3,4-*d*]Pyrimidine Derivative Si306 Encapsulated into Anti-GD2-Immunoliposomes as Therapeutic Treatment of Neuroblastoma

**DOI:** 10.3390/biomedicines10030659

**Published:** 2022-03-12

**Authors:** Enrico Rango, Fabio Pastorino, Chiara Brignole, Arianna Mancini, Federica Poggialini, Salvatore Di Maria, Claudio Zamperini, Giulia Iovenitti, Anna Lucia Fallacara, Samantha Sabetta, Letizia Clementi, Massimo Valoti, Silvia Schenone, Adriano Angelucci, Mirco Ponzoni, Elena Dreassi, Maurizio Botta

**Affiliations:** 1 Dipartimento Biotecnologie, Chimica e Farmacia, Università degli Studi di Siena, 53100 Siena, Italy; rango.enrico@gmail.com (E.R.); arianna.mancini3@gmail.com (A.M.); federicapoggialini91@gmail.com (F.P.); dimaria6@student.unisi.it (S.D.M.); iovenitti.giulia@gmail.com (G.I.); al.fallacara@gmail.com (A.L.F.); botta.maurizio@gmail.com (M.B.); 2 Laboratory of Experimental Therapies in Oncology, IRCCS Istituto G. Gaslini, 16148 Genoa, Italy; chiarabrignole@gaslini.org (C.B.); mircoponzoni@gaslini.org (M.P.); 3 Lead Discovery Siena S.r.l., Via Vittorio Alfieri 31, 53019 Castelnuovo Berardenga, Italy; claudiozamperini@gmail.com; 4 Dipartimento Scienze Cliniche Applicate e Biotecnologiche, Università dell’Aquila, Via Vetoio, 67100 Coppito, Italy; samantha.sabetta@graduate.univaq.it (S.S.); letizia.clementi@graduate.univaq.it (L.C.); adriano.angelucci@univaq.it (A.A.); 5 Dipartimento Scienze della Vita, Università degli Studi di Siena, Via Aldo Moro 2, 53100 Siena, Italy; massimo.valoti@unisi.it; 6 Dipartimento di Farmacia, Università degli Studi di Genova, Viale Benedetto XV 3, 16132 Genoa, Italy; schenone@difar.unige.it; 7 Center for Biotechnology, Sbarro Institute for Cancer Research and Molecular Medicine, College of Science and Technology, Temple University, BioLife Science Building, Suite 333, 1900 North 12th Street, Philadelphia, PA 19122, USA

**Keywords:** neuroblastoma, c-Src inhibitor, liposomes, anti-GD2 monoclonal antibody, immunoliposomes

## Abstract

Si306, a pyrazolo[3,4-*d*]pyrimidine derivative recently identified as promising anticancer agent, has shown favorable in vitro and in vivo activity profile against neuroblastoma (NB) models by acting as a competitive inhibitor of c-Src tyrosine kinase. Nevertheless, Si306 antitumor activity is associated with sub-optimal aqueous solubility, which might hinder its further development. Drug delivery systems were here developed with the aim to overcome this limitation, obtaining suitable formulations for more efficacious in vivo use. Si306 was encapsulated in pegylated stealth liposomes, undecorated or decorated with a monoclonal antibody able to specifically recognize and bind to the disialoganglioside GD2 expressed by NB cells (LP[Si306] and GD2-LP[Si306], respectively). Both liposomes possessed excellent morphological and physio-chemical properties, maintained over a period of two weeks. Compared to LP[Si306], GD2-LP[Si306] showed in vitro specific cellular targeting and increased cytotoxic activity against NB cell lines. After intravenous injection in healthy mice, pharmacokinetic profiles showed increased plasma exposure of Si306 when delivered by both liposomal formulations, compared to that obtained when Si306 was administered as free form. In vivo tumor homing and cytotoxic effectiveness of both liposomal formulations were finally tested in an orthotopic animal model of NB. Si306 tumor uptake resulted significantly higher when encapsulated in GD2-LP, compared to Si306, either free or encapsulated into untargeted LP. This, in turn, led to a significant increase in survival of mice treated with GD2-LP[Si306]. These results demonstrate a promising antitumor efficacy of Si306 encapsulated into GD2-targeted liposomes, supporting further therapeutic developments in pre-clinical trials and in the clinic for NB.

## 1. Introduction

Neuroblastoma (NB) is a solid tumor that occurs in the pediatric population and causes 15% of childhood cancer deaths [1]. It is an extracranial tumor that originates from the primitive sympathetic ganglia and that is principally found in the adrenal glands, the thorax, the abdomen, and the pelvic sympathetic ganglia [2]. The severity of the tumoral manifestation together with its biological characteristics allows for the classification of NB into three categories, differing each other for prognosis and genetic alterations. The most severe form of NB, high-risk NB, is characterized by the loss of TRK-A expression and the allelic 1p chromosome, N-myc amplification, and poor prognosis [3,4,5]. It is treated with surgery, radiotherapy, immunotherapy, stem cells transplantation and chemotherapy, which are not sufficient to increase the children’s survival. In addition, NB often develops resistance mechanisms that make drugs ineffective. For these reasons, and to avoid the toxic effects of the therapies, it is urgent to investigate novel therapeutic strategies to be used for the treatment of high-risk NB [6,7].

**Figure 1:**
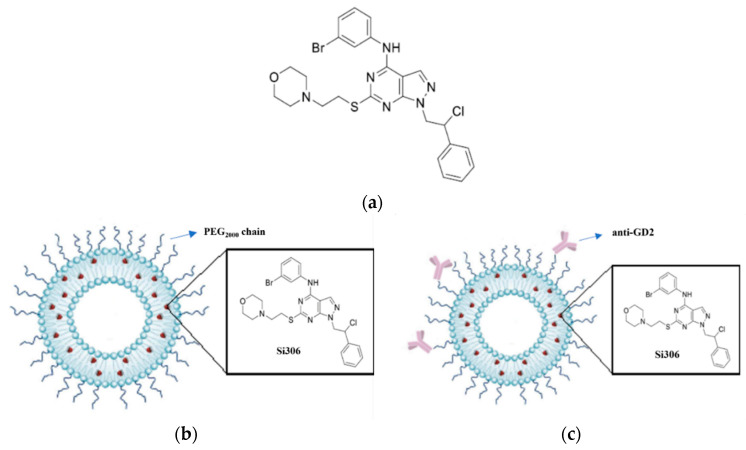
Development of Si306-loaded, untargeted and GD2-targeted pegylated stealth liposomes. (**a**) Chemical structure of Si306; schematic representation of (**b**) LP[Si306] and (**c**) GD2-LP[Si306].

Compound Si306 (Figure 1a), a pyrazolo[3,4-*d*]pyrimidine derivative has been identified as a potent inhibitor of c-Src tyrosine kinase (Ki equal to 0.13 μM), which belongs to the Src family kinases [8]. c-Src activity is elevated in several types of human cancer. Its role in tumor progression, maintenance and survival has been associated to several solid and hematologic cancers, such as colon [9], breast [10], lungs [11], liver [12], prostate [13], pancreatic [14], glioblastoma multiforme (GBM) [15,16], NB [17], chronic myelogenous leukemia [18], and lymphomas [19].

As a potent inhibitor of c-Src, Si306 was found to induce apoptosis and to reduce proliferation in GBM [20,21,22,23] and NB [8,24,25] tumor cell lines, while negligible activity was evidenced in non-tumoral cells [8]. In vivo, an oral administration of Si306 showed a delay of tumor growth in a subcutaneous NB animal model [8]. However, the limitations to the further development of this promising compound are represented by its low water solubility and its limited ability to be formulated and administered parenterally [8]. In this scenario, the development of nanoparticles as drug delivery systems may be a promising solution. In particular, due to unique characteristics, such as biocompatibility and biodegradability, liposomes have shown enormous potential as drug carriers for cancer therapy [26,27,28]. Liposomal vehicles are also known to overcome low “drug” solubility limitations, to extend the circulation time of the encapsulated “drugs” in the blood stream, to reduce drug clearance, and to decrease off-targets side effects [29,30,31,32]. In addition, due to the disorganization of the fenestrated tumor vascular endothelium, the use of liposomes as drug delivery systems allows the passive targeting of the transported compound to the tumor site, thanks to the EPR (enhanced permeability retention) effect [33,34]. To date, several clinical trials involve the use of liposomal suspensions containing both hydrophilic and hydrophobic chemotherapeutics [28]. At the same time, tumor targeting can be further ameliorated by decorating the outer surface of nanocarriers with a ligand (i.e., peptides, carbohydrates, and antibodies) that can specifically bind to the receptors expressed on the tumor cell membrane. Among these, antibody-conjugated liposomes (called immunoliposomes), which can target antigen-expressing tumor cells, have attracted considerable attention as targeted therapies due to their ability to selectively deliver the encapsulated drug to tumor cells, improving efficacy and reducing toxicity [35]. In this context, the disialoganglioside GD2 expressed by tumors of neuroectodermal origin (e.g., melanoma and NB [36]), and very limited expression on healthy cerebellum and peripheral nerves [37,38], represents a well-established target for the design of an active targeting strategy for NB [39,40,41]. In particular, liposomes conjugated with the whole or the Fab fragments of anti-GD2 antibody have been reported for the targeted delivery of doxorubicin [42,43], fenretinide [44], antisense oligodeoxynucleotides [45], and siRNAs/miRNAs [46,47,48] in NB.

The antitumor effectiveness of Si306, either free or encapsulated in untargeted (LP[Si306]) or GD2-targeted (GD2-LP[Si306]) liposomes (Figure 1b,c) was here evaluated both in vitro and in a clinically relevant mouse model of NB [49].

## 2. Materials and Methods

### 2.1. Drugs and Materials

We synthesized and characterized Si306 [21]. Anti-GD2 monoclonal antibody was purchased from Polo GGB—Siena, Italy. All reagents, solvents and materials were purchased from Sigma Aldrich Srl (Milan, Italy). Phospholipids used for the preparation of liposomes were purchased from Avanti Polar Lipids (Avanti Polar Lipids, Inc., Alabaster, AL, USA). The RPMI-1640 and Dulbecco’s Modified Eagle’s Medium (DMEM) culture media, foetal bovine serum (FBS), L-glutamine and Penicillin-Streptomycin were purchased from Euroclone S.p.A. (Milan, Italy).

### 2.2. Cells Lines and Animal Models

Human neuroblastoma (NB) cell lines (IMR-32, HTLA-230, SK-N-AS and SH-SY5Y) were grown in complete DMEM medium, as previously described [50]. In some experiments, IMR-32 cells were infected with retrovirus expressing the firefly luciferase (luc) gene, as previously reported [51]. Luciferase activity of IMR-32-luc cells was confirmed by bio-luminescent imaging (BLI, Lumina-II, Caliper Life Sciences, Hopkinton, MA, USA) after a 10 min incubation with 150 μg/mL d-luciferin (Caliper Life Sciences) diluted in cell culture medium, as previously described [48,50]. Normal human dermal fibroblasts (Fibro 2–93) were purchased from American Type Culture Collection (ATCC, Manassas, VA, USA) and grown in complete RPMI-1640 medium, as previously described [50]. Cells were tested for mycoplasma contamination, characterized by cell proliferation and morphology evaluation, and authenticated at time of experimentation by multiplex STR-profiling test (PowerPlex^®^ Fusion, Promega, Milan, Italy) by BMR Genomics (Padova, Italy) and validated using ATCC STR, DSMZ STR and NCBI databases.

Naive BALB/c male mice (aged 4–6 weeks, Charles Rivers—Milan, Italy) were used for pharmacokinetic (PK) and biodistribution (BD) studies. Mice were maintained under pathogen-free conditions and given food and water at libitum. All of the procedures used on these animals were approved by Institutional Animal Use and Care Committee at Università degli Studi di Siena and authorized by the Italian Ministry of Health, according to Legislative Decree 116/92, which implemented the European Directive 86/609/EEC on laboratory animal protection in Italy (*n*. 412/2016-PR). Methods for all of the conducted experiments were performed in accordance with regulations, standards, and guidelines of the Animal Use and Care Committee of Università degli Studi di Siena.

Female athymic Nude-Foxn1^nu^ (nu/nu) tumor-bearing mice (Envigo, Bresso, Italy) were used for PK and tumor homing studies, and for therapeutic experiments in NB tumor-bearing animals. Specifically, IMR-32 (wild-type and luc-transfected) cells (1 × 10^6^ cells in 10 μL culture medium) were inoculated in the left adrenal gland of five-week-old nu/nu mice, as described [50]. No mice died as a result of the surgery. Mice body weight and general physical status were daily recorded. When any sign of discomfort or poor health arose (i.e., abdominal dilatation, dehydration, paraplegia, >20% weight loss) mice were anaesthetized with xilezine (Xilor 2%, Bio98 Srl, Milan, Italy) and euthanized by CO_2_ inhalation. The day of euthanasia was recorded as the day of death. In accordance with the 3Rs policy, experiments were reviewed and approved by the licensing and ethical committee of Ospedale Policlinico San Martino in Genoa, and by the Italian Ministry of Health (*n*. 661/2016-PR).

### 2.3. Preparation of Si306-Containing Liposomes

Liposomes and anti-GD2-immunoliposomes, loaded with Si306, (LP[Si306]) and GD2-LP[Si306], respectively) were prepared using the thin layer evaporation method [52]. Specifically, they were composed of phospholipids (PLs) DPPC (1,2-dipalmitoyl-sn-glycero-3-phosphocholine), POPC (1-palmitoyl-2-oleoyl-sn-glycero-3-phosphocholine), DPPE-PEG_2000_ (N-(Carbonyl-methoxypolyethylenglycol2000)-1,2-dipalmitoyl-sn-glycero-3-phosphoethanolamine sodium salt) (molar ratio 0.3:0.7:0.04), and DPPC, POPC, DPPE-PEG_2000_ and DSPE-PEG_2000_-Maleimide (molar ratio 0.3:0.7:0.04:0.006), respectively. Si306 and cholesterol (15% of the total moles of DPPC, POPC and DPPE-PEG_2000_) were dissolved, together with the PLs, in a mixture of chloroform/methanol (3:1 *v*/*v*) and transferred into a round-bottom flask. The organic solvents were removed by rotary evaporation under vacuum at 37 °C and then under nitrogen flow to remove any remaining solvent residues. The dried lipid film obtained was then hydrated in 25 mM HEPES and 140 mM NaCl buffer, pH 7.4 (HEPES buffer) under continuous mechanical agitation at 60 °C. The liposomal suspension was extruded at a temperature of 60 °C through polycarbonate membranes with a pore size of 0.2 µm (5 passages) first and then of 0.1 µm (10 passages) to obtain unilamellar vesicles. Residues of not encapsulated Si306 were removed from the liposomes by size-exclusion chromatography, passing the liposomal suspension through a Sephadex G-25 column in HEPES buffer, pH 7.4. Finally, drug encapsulation efficacy (%EE) and amount of cholesterol were determined by UV/LC-MS analysis using Agilent 1100 LC/MSD VL system (G1946C) (Agilent Technologies, Palo Alto, CA, USA) constituted as previously described [22]. Chromatographic analyses for drug encapsulation efficacy in the liposomal formulations, and in general, the quantifications of Si306 were performed using a Phenomenex Kinetex EVO C18-100Å (150 × 4.6 mm, 5 μm particle size) at room temperature, at flow rate of 0.6 mL/min, and injection volume of 20 μL, operating with a gradient elution of A: water (H_2_O) and B: acetonitrile (ACN): t = 0 min 5% of B, t = 1 min 5% of B, t = 10 min 95% of B and kept up to 19 min, t = 20 min 5% of B. Both solvents were acidified with 0.1% *v*/*v* of formic acid. UV detection was monitored at 254 nm. Cholesterol quantification, required during the preparation of liposomal formulations, were performed using the same chromatographic column, operating with an isocratic elution of methanol (MeOH) for 10 min. UV detection was monitored at 210 nm. All quantification analyses were performed by reference to the appropriate calibration curve.

In the anti-GD2-immunoliposomes preparation, unilamellar vesicles with maleimide-terminated polyethylene glycol-DSPE chains were obtained. To couple the anti-GD2 antibody to the maleimide terminus, anti-GD2 (previously concentrated from 2.5 to 8–10 mg/mL, using Amicon filters 30 K—Sigma Aldrich Srl, Milan, Italy) was firstly functionalized via Traut’s reagent (2-iminothiolane, 25 mM) at a molar ratio of 20:1 (2-iminothiolane:mAb) for 1 h at room temperature in HEPES buffer pH 8.0. Unreacted Traut’s reagent was removed via size-exclusion chromatography using Bio-Gel P-6 desalting Cartridge column in HEPES buffer pH 8. The quantification of activated anti-GD2 was performed by using Bradford protein quantification assay. In parallel, performing Ellman assay, 1 free-SH group per antibody has been estimated. The coupling reaction was finally run at a molar ratio of 1:4500 (anti-GD2:PLs), for 16 h at 5 °C, in continuous slow magnetic stirring. Uncoupled mAb molecules were separated from the liposome suspension passing the coupling mixture through a Sepharose CL-4B column in HEPES buffer (pH 7.4), as previously described [42]. The quantification of the coupled anti-GD2 was performed by using bicinchoninic acid (BCA) assay then quantification of Si306 and cholesterol were determined as described above.

### 2.4. Characterization of Liposomal Formulations

Liposomes and immunoliposomes have been fully characterized by evaluating their physiochemical properties, morphology, amount of PLs (by quantifying cholesterol measure by UV/LC-MS) and drug encapsulation efficiency. Specifically, particle size diameter (in nm), polydispersity index (PDI) and ζ-potential (in mV) of both liposomal formulations were analyzed by dynamic light scattering (DLS), using the particle size analyzer Zeta Sizer Nano ZS90 (Malvern Instruments), as previously reported [48]. All of the results are expressed as mean values ± S.D. calculated from three independent experiments. Liposome morphology was observed by cryo-TEM. Briefly, 3 μL of the sample were applied on Quantifoil^®^ holey carbon grids and then frozen in liquid ethane to achieve sample vitrification. Frozen samples were stored in liquid nitrogen until EM imaging. Vitrified samples were imaged using a CM200 FEG transmission EM. EM images were acquired at 27,500× magnification (pixel size 0.602 nm) at −12, −18 μm defocus. Drug Encapsulation Efficiency percentage (EE%) was determined by UV/LC-MS analysis. Liposomal suspension aliquots were treated with ethanol in 1:10 *v*/*v* ratio to extract Si306, which was quantified by UV/LC-MS by reference to the appropriate calibration curve. EE% was measured with respect to the amount of compound initially added to the organic solution, using the following equation:

EE% = (mg of encapsulated drug/mg of total drug) × 100

The quantification of the anti-GD2 antibody conjugated on the immunoliposome surfaces was performed by using QuantiPro™ BCA Assay Kit (Sigma Aldrich Srl—Milan, Italy).

### 2.5. STABILITY and In Vitro Si306 Release Studies

Both liposomal formulations were stored at 4 °C for 2 weeks. The samples were evaluated over time by DLS measurements for changes in particle size, ζ potential and PDI. Si306 release from liposomes was evaluated by dialysis. Specifically, liposomal samples have been sealed in a dialysis bag (cut off 10 kDa) and dialyzed against 20 mL of PBS pH 7.4, 50 mg/mL of BSA (Bovine serum albumin at physiological plasma concentration). The entire release medium was gently stirred at 37 °C. At predetermined time intervals (0, 1, 2, 4, 6, 24, 48, 72 and, 96 h), 0.5 mL of release medium was collected and complemented with fresh PBS at the same temperature. The sample solution was treated with 1.5 mL of acetonitrile (ACN) and centrifuged at 5000 rpm for 20 min. The supernatant obtained was dried under nitrogen, re-suspended in 0.1 mL of methanol, and analyzed by UV/LC-MS.

### 2.6. Cellular Association of Anti-GD2-Targeted Immunoliposomes

To study the cellular association of untargeted or GD2-targeted LP[Si306], 0.1 mol% of the fluorescent lipid, 1,2-dioleoyl-sn-glycero-3-phosphoethanolamine-N-(carboxyfluorescein) ammonium salt (PE-CF), was added during the lipid thin layer preparation. Cellular association of Si306-containing liposomes was assessed by flow cytometry (FCM; FacsCalibur, Becton-Dickinson Immunocytometry Systems) Becton-Dickinson, Italia; Milan, Italy [53]. Briefly, one × 10^6^ HTLA-230, IMR-32, SK-N-AS and SH-SY5Y NB cells were incubated for 1 h at 4 °C with PE-CF-labelled untargeted or GD2-targeted LP[Si306]. Samples were subsequently washed with PBS, and fluorescence enumerated by FCM. The results are expressed as mean ratio fluorescence intensity (MRFI) calculated as the MFI of samples incubated with both liposomes, divided by the MFI of control (CTR, no liposomes) cells.

### 2.7. Western Blot Analysis

Protein expression was determined in total cell lysates by Western blot analysis of IMR-32, HTLA-230 and SH-SY5Y NB cells using primary anti-Src (36D10), anti-pSrc (D49GA), and anti-GAPDH antibodies (Abs, Cell Signaling Technology, Danvers, MA, USA), according to the manufacturer’s suggested dilution. The secondary Ab used, HRP-conjugated anti-rabbit IgG, was purchased from Cell Signaling and used according to the manufacturer’s suggested dilution. Briefly, cells were lysed by adding RIPA buffer (150 mM NaCl, 1% Triton X-100, 0.5% Na deoxycholate, 0.1% SDS, 50 mM Tris HCl at pH 8.0) plus protease and phosphatase inhibitors (Merck KGaA, Darmstadt, Germany). Protein concentration was determined spectrophotometrically by Bradford colorimetric assay (SERVA Electrophoresis GmbH). Thirty-five micrograms of electrophoretically resolved proteins in 10% SDS-polyacrylamide gels were electro-transferred onto 0.2 m Amersham protran nitrocellulose membrane (Cytiva Europe GmbH, Freiburg im Breisgau, Germany) for 90 min at 350 mA. After transfer, the membrane was blocked with 10% fat-free milk blocking solution in TBS-T (Tris Buffered Saline with Tween 20), incubated with the primary antibody at 4 °C overnight, and incubated with the HRP-conjugated secondary antibody for 1 h. Following two 10-min washes in TBS-T and two final 10-min washes in TBS the membrane was incubated in ECL solution (Euroclone, Milan, Italy) for approximately 2 min. Chemiluminescent signal was acquired by the Chemidoc XRS system and digitally processed with Imagelab software (Bio-Rad Laboratories Inc.; Hercules, CA, USA).

### 2.8. In Vitro Cytotoxicity Studies

Cytotoxicity experiments were performed to evaluate the effectiveness of Si306, either free (dissolved in DMSO) or encapsulated into untargeted and GD2-targeted liposomes [54]. Briefly, IMR-32, HTLA-230 and SH-SY5Y cells were seeded in 96-well plates (8 × 10^3^ cells/well). Human fibroblasts were used as GD2-negative healthy control cells (5 × 10^3^ cells/well). The day after seeding, cells were incubated with increasing concentration of Si306 (0.1, 1.0, 10.0 and, 100.0 μM) either free or liposomes-encapsulated, for 24 h at 37 °C. Cells were then washed twice with PBS before replacing them with fresh complete medium and incubating for an additional 72 h (total incubation time equal to 96 h). At the end of the incubation, the MTT (Sigma) assay was performed. The tetrazolium dye was added, then plates were incubated for 3–4 h at 37 °C; formazan crystals were then dissolved in acid isopropanol, and then the optical density of the stained solution was quantified by the use of a Multiskan™ FC Microplate Photometer (Thermo Fisher Scientific Italia, Monza, Italy) at the dual wavelengths of 570 and 650 nm [54].

### 2.9. Pharmacokinetic, Biodistribution and Tumor Uptake Studies

For PK, BD and tumor uptake studies, naive BALB/c (*n* = 5/group) or NB-bearing nude (*n* = 3/group) mice were injected via the tail vein with a single dose of 5 mg/kg of Si306, either free, dissolved in a mixture of Tween80 (10% *v*/*v*), benzyl alcohol (1% *v*/*v*) and a 10 mM solution of citric acid, as previously described [22], or encapsulated into untargeted and GD2-targeted liposomes. At several time points (0.08, 0.25, 0.5, 1, 2, 4, 8, 24, 48 h), post-injection, mice (*n* = 5/group) were treated intraperitoneally with heparin (5000 U/kg) and euthanized under CO_2_. Blood, liver, spleen, kidneys and tumor tissues were collected and processed as reported [22,55]. The quantifications of Si306 in blood, organ and, tumor samples were performed by LC-MS/MS analysis using a HPLC Agilent 1200 Series (Agilent Technologies, Italia, SpA, Milan, Italy) coupled with a mass spectrometer TSQ Quantum Access (Thermo Fisher Scientific Italia, Monza, Italy), equipped with electrospray ion source (ESI) and triple quadrupole analyzer. The Xcalibur software (Thermo Fisher Scientific) was available for managing the instrument, collecting, and analyzing data. The ESI-MS conditions were optimized through the direct injection of a Si306 standard solution (as well as for Si34 used as internal standard), in negative ion current mode, using nitrogen as atomizing gas. The transitions as well as the capillary voltage and the collision energy used are appropriated for each tested compound. Preliminarily, to identify the analytes and relevant retention times, selected samples were analyzed on TIC mode (total ion current) in the range of 300–1000 *m*/*z*. Then the quantification of selected species was carried out via SIM (single ion monitoring) method. Chromatographic separation was obtained using a Phenomenex Kinetex C18-100Å column (30 × 2.1 mm) with 2.6 μm particle size (bearing a guard column Phenomenex SecurityGuard™ ULTRA Holder) and operating with a gradient elution of A: water (H_2_O) and B: acetonitrile (ACN): t = 0 min 5% of B, t = 1 min 5% of B, t = 3 min 95% of B and kept up to 9 min, t = 10 min 5% of B. Both solvents were acidified with 0.1% *v*/*v* of formic acid. The flow rate was 0.2 mL/min, and the injection volume was 5 μL. The analytical quantifications were performed by comparison with appropriate calibration curves using Si34 as internal standard, and calibration curves were acquired daily (in duplicates). In addition, the possible of the presence or absence of matrix effect and the recovery of extracted Si306 were also evaluated [56,57] (see Supplementary Materials for more details). The PK parameters were calculated by non-compartmental analysis using PKSolver software [22,55].

### 2.10. In Vivo Therapeutic Experiments

In the first set of therapeutic experiments, tumor growth and therapy response were followed by BLI imaging. Specifically, seven days after IMR-32-luc cell inoculation, mice were randomly assigned to different groups after BLI evaluation (*n* = 12/group) and i.v. treated 5 mg/kg of Si306, either dissolved in a mixture of Tween80 (10% *v*/*v*), as reported above, or encapsulated in LP (LP[Si306]) or GD2-LP (GD2-LP[Si306]). The amount of Si306 mixed into the lipidic mixture during the liposomes preparation produced a final concentration, over lipid suspension, of 0.5 mg/mL. The treatment was performed every three days, for eight times total.

In the second set of the therapeutic studies, the survival time represented the main criterion for evaluating the tumor response to treatment. Here, in order to try increasing the amount of Si306 potentially delivered to the tumor, the amount of Si306 mixed into the lipidic mixture during the liposomes preparation was increased, producing a final concentration, over lipid suspension, of 2.5 mg/mL. Seven days after IMR-32 cell inoculation, mice were randomly assigned to the different experimental groups (*n* = 7/group) as above, and i.v. treated with 25 mg/kg of Si306, following the schedule plan used for the imaging study. 25 mg/kg of Si306, already used in vivo as free compound without signs of toxicity [22], was the maximum dosage achieved, after the encapsulation of increased amounts of Si306 into the lipidic mixture. In all of the in vivo experiments, control mice (CTR) received HEPES-buffered saline. Mice were weighed 24 h after each treatment to evaluate possible macroscopic toxicity.

### 2.11. Immunohistochemistry Studies

In the first therapeutic experiment, *n* = 5 out of 12 mice/group were anaesthetized with xilezine and euthanized 24 h after the fifth treatment. The tumors were harvested, fixed in 4% formaldehyde in 0.1 M phosphate buffer, pH 7.2 and embedded in paraffin for subsequent immunohistochemical analyses. Specifically, slide-mounted tissue sections (4-μm thick) were deparaffinized in xylene and serially hydrated in 100%, 95%, and 80% ethanol. Endogenous peroxidases were quenched in 3% H_2_O_2_ in phosphate-buffered saline (PBS) for 1 h. Then slides were incubated with anti-human primary antibodies (10 μg/mL) for 1 h and, subsequently, with peroxidase-conjugated secondary antibody for 30 min at room temperature. Antibody binding was revealed using the Sigma fast 3,30-diaminobenzidine tablet set (Sigma). Counterstaining was performed using hematoxylin solution. Anti-Src (36D10), anti-pSrc (D49GA) and secondary antibodies were purchased from Cell Signaling Technology (Danvers, MA, USA). The expression of Src and p-Src was quantified using digitally acquired images and ImageJ Fiji software (Johannes Schindelin, Albert Cardona, Mark Longair, Benjamin Schmid, and others, https://imagej.net/Fiji/Downloads, version 1.2; access date from January to June 2021. Software elaboration of images was performed according to previous protocol [58]. The final score was calculated by multiplying the stain intensity by the number reflecting the percentage of positive tumor cells (0, no positive cells; 1, <10%; 2, 10–50%; 3, 51–80%; or 4, >80%).

### 2.12. Statistics

All of the in vitro experiments were performed at least three times with similar results. Each experimental condition, for the assays performed in 96-well plates, was carried out in quadruplicate”. The analyses were performed with Prism 5 software (GraphPad, La Jolla, CA, USA): one-and two-way analyses of variance (ANOVA) with Tukey’s Multiple Comparison Test were used to evaluate differences within treatments. Survival curves were drawn as Kaplan-Meier Cumulative Proportion Surviving graphs, and corresponding *p*-values were calculated by the use of the log-rank (Mantel-Cox) test. Asterisks indicate the following *p*-value ranges: * *p* < 0.05, ** *p* < 0.01, *** *p* < 0.001.

## 3. Results

### 3.1. Characterization of Liposome Formulations

Untargeted and GD2-targeted liposomes, (LP[Si306] and GD2-LP[Si306], respectively) have been characterized in terms of mean diameter, ζ-potential and polydispersity index (PDI). As reported in Table 1, the size and ζ-potential values were 126 ± 15 nm and −22.6 ± 3.5 mV for LP[Si306], and 133 ± 16 nm and −19.3 ± 1.6 mV for GD2-LP[Si306].

**Figure 2:**
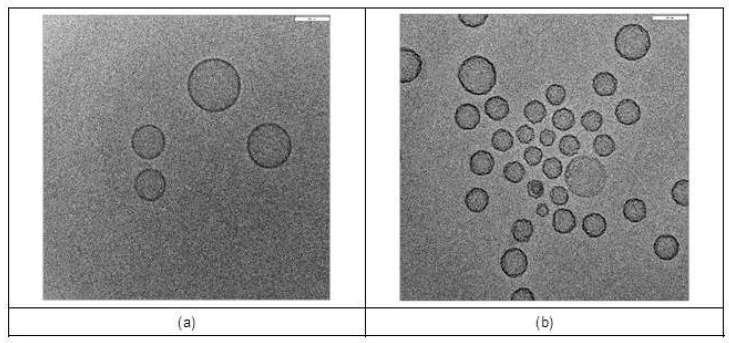
Representative Cryo-TEM micrographs of Si306-loaded liposomes. Images of (**a**) LP[Si306] and (**b**) GD2-LP[Si306], obtained by Cryo-TEM analysis. Bar: 80 nm.

The amount of phospholipid (PLs), determined as a function of cholesterol levels, was 9.06 ± 0.65 μmol/mL for LP[Si306] and 10.80 ± 1.84 μmol/mL for GD2-LP[Si306]. Mean drug encapsulation efficiency percentage (EE%) was more than 70% for both formulations (Table 1). In addition, the liposomes morphology and particle size have been evaluated using Cryo Transmission Electron Microscopy (Cryo-TEM). As shown in Figure 2a,b, the Cryo-TEM analysis confirmed the presence of a homogeneous and unilamellar population of liposomal nanoparticles with particle sizes ranging from 90–120 nm and 80–130 nm for LP[Si306] and GD2-LP[Si306], respectively.

The average thickness of the phospholipidic bilayer was 7.02 nm for LP[Si306] and 7.21 nm for GD2-LP[Si306]. Moreover, a surface roughness has been noted on GD2-LP[Si306], which support the presence of antibodies on their surface (Figure 2a). The levels of coupled anti-GD2 antibody were estimated in a range of 8–14 μg/μmol of PLs.

### 3.2. Stability Studies and In Vitro Release

To determine whether LP[Si306] and GD2-LP[Si306] were stable at 4 °C, liposomal suspensions were stored over a period of two weeks and periodically sampled to measure mean particle size, ζ-potential and PDI. The stability tests for both formulations are summarized in Table 2.

**Figure 3:**
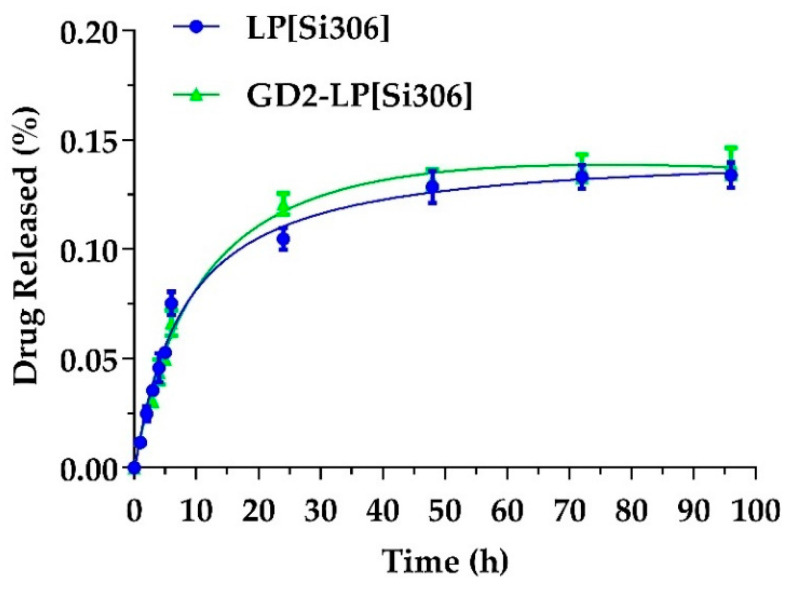
In vitro release of Si306-loaded untargeted (blue curve) and GD2-targeted (green curve) liposomes.

The slight modifications observed over time indicate that these liposomes warrant features of a proper rate of stability, thus representing formulations suited for in vivo studies. Furthermore, both liposomal formulations showed minimal drug leakage, retaining >95% of the encapsulated compound over a period of 96 h (Figure 3).

### 3.3. Cellular Association of Anti-GD2-Targeted Immunoliposomes

**Figure 4:**
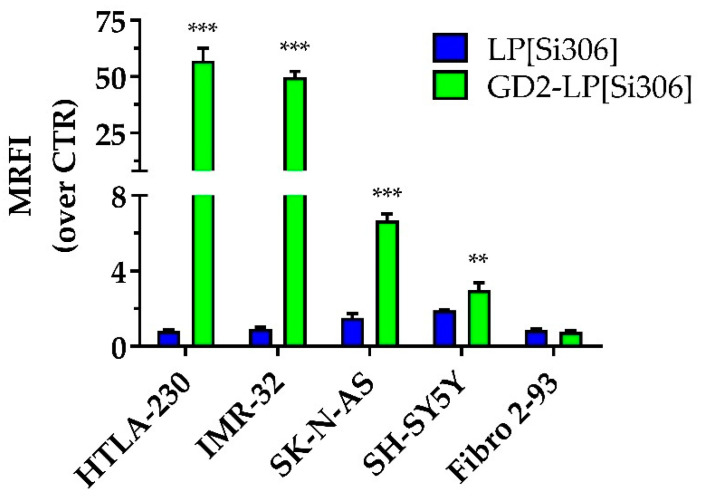
Cellular association of untargeted and GD2-targeted liposomes on neuroblastoma cell lines. Neuroblastoma cells (HTLA-230, IMR-32, SK-N-AS, and SH-SY5Y) and healthy fibroblasts (Fibro 2–93) were incubated at 4 °C, for 1 h, with Carboxy fluorescein-labelled liposomes at 400 nmol phospholipid/mL concentration. Fluorescence associated with cells was evaluated by flow cytometry. Binding is expressed as mean relative fluorescence intensity (MRFI) normalized over control cells (no liposomes incubation). Columns: MRFI ± SD. ** *p* ˂ 0.01: GD2-LP[Si306] vs. LP[Si306]; *** *p* ˂ 0.001: GD2-LP[Si306] vs. LP[Si306].

The levels of cellular association of carboxyfluorescein-labeled LP[Si306] and GD2-LP[Si306] were evaluated by FCM on four human NB cell lines (HTLA-230, IMR-32, SK-S-AS and SH-SY5Y), expressing different levels of GD2, and on human healthy GD2-negative fibroblasts (Fibro 2–93), used as control. After 1 h of incubation at 4 °C, the cellular association of GD2-LP[Si306] was significantly higher compared to the untargeted formulation (Figure 4).

This binding was dependent on the GD2 expression of the different cell lines used. As a consequence, negligible cellular association was seen in GD2-negative fibroblasts (Figure 4).

### 3.4. In Vitro Cytotoxicity Studies

After the demonstration by western blot analyses of the constitutive expression of both c-Src and its phosphorylated form (see Figure S1 in the Supplementary Materials), both representing the pharmacological targets of Si306, the cytotoxic effects of Si306, either free or encapsulated in untargeted or GD2-targeted liposomes, were tested against HTLA-230, IMR-32 and SH-SY5Y NB cell lines. As summarized in Table 3, GD2-LP[Si306] were more cytotoxic than Si306 as free drug and encapsulated in untargeted liposomes, against all of the cell lines tested. To note, the antitumor effect was correlated to the cellular association results obtained (Figure 4), as a consequence of the degree of expression of the receptor GD2 [59].

### 3.5. Pharmacokinetic and Biodistribution Studies on Healthy Mice

**Figure 5:**
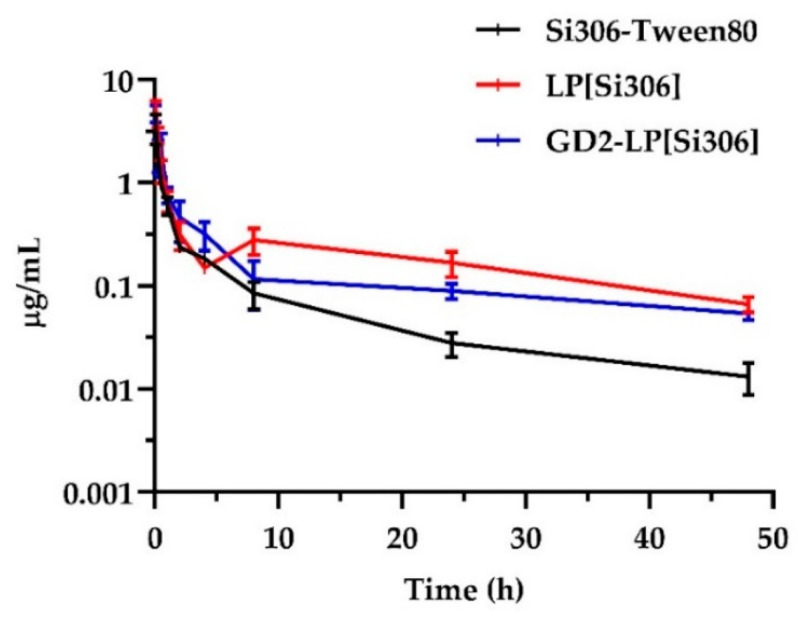
Pharmacokinetic profiles of Si306 in vivo. Plasma concentration-time curves (mean ± S.E.M., *n* = 5) in healthy mice, after i.v. administration of a single dose of 5 mg/kg Si306, either free (Si306-Tween80) or encapsulated into untargeted and GD2-targeted liposomes (LP[Si306] and GD2-LP[Si306], respectively). The plasma concentration in the y-axis is expressed as log_10_ scale.

As previously shown, long circulation times are required for nanoparticles to reach the tumor sites [32,60]. Here, the quantification of Si306, either free (Si306-Tween80) or encapsulated in liposomal formulations, remained in blood and organs after systemic administration, was performed by LC-MS/MS. As shown in Figure 5, the plasma concentration-time curves and the corresponding PK parameters (Table 4) indicate that free Si306 was removed more rapidly than Si306 encapsulated in liposomes, indicating that liposomes have good stability and long circulation times.

Specifically, Si306 encapsulated in liposomes showed higher C_max_ values (5.42 and 4.73 µg/mL for LP[Si306] and GD2-LP[Si306], respectively) than the free drug (3.42 µg/mL). Moreover, both LP[Si306] and GD2-LP[Si306] showed values of AUC_0__→48h_ and MRT higher than Si306-Tween80, while the plasma clearance (CL) values confirm the slower elimination of the drug from the circulatory stream when delivered by liposomes (Table 4).

The BD of Si306 was evaluated in the liver, spleen, and kidneys. In all tissues analyzed, an immediate distribution followed by a concentration decrease in a time-dependent manner was observed (see Figure S2a,c in the Supplementary Materials). Compared to Si306 administered in free form, both liposomal formulations showed, as expected, higher values of AUC_0__→48h_ in the liver and spleen (Table S1), RES-rich organs, site of action of the mononuclear phagocyte system [61].

### 3.6. PK and Tumor Uptake in NB-Bearing Mice

**Figure 6:**
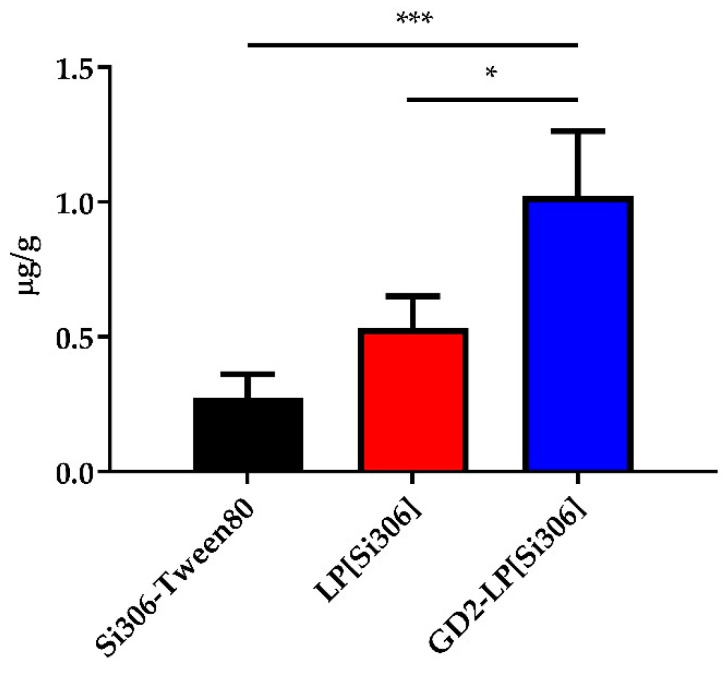
In vivo Si306 tumor uptake. IMR-32-bearing mice (*n* = 3/group) were treated with 5 mg/kg Si306, either free (Si306-Tween80) or encapsulated into untargeted and GD2-targeted liposomes (LP[Si306] and GD2-LP[Si306], respectively). 24 h after treatments, mice were euthanized, tumors removed and processed as described in the Materials and Methods section. Columns: mean ± SD. * *p* ˂ 0.05: GD2-LP[Si306] vs. LP[Si306]; *** *p* ˂ 0.001: GD2-LP[Si306] vs. Si306-Tween80.

The PK study was also performed in an orthotopic mouse model of NB, as previously described [43]. Mice were injected in the adrenal gland with 1 × 10^6^ IMR-32 cells and i.v. treated 1 week after NB cells challenge, with 5 mg/kg of Si306, either free (Si306-Tween80) or encapsulated in LP and GD2-LP. Plasma concentration-time curves and PK parameters are shown in Figure S3 and Table S2, respectively. Plasma AUC_0__→48h_ values confirmed the results obtained in healthy mice: both liposomal formulations allow for a longer plasma circulation of Si306 with AUC_0__→48h_ values 5- and 7-fold (for LP[Si306] and GD2-LP[Si306], respectively) higher than the drug injected as in free form. Finally, tumor accumulation experiments indicated that, the uptake in the tumor masses 24 h after the injection of the c-Src inhibitor, was significantly superior in NB-bearing mice receiving Si306 encapsulated into GD2-LP[Si306], compared with that obtained in mice treated with either free Si306, or Si306 encapsulated in untargeted LP (Figure 6), indicating the importance of the active targeting, via the GD2 receptor, for the subsequent therapeutic experiments.

### 3.7. In Vivo Therapeutic Studies

**Figure 7:**
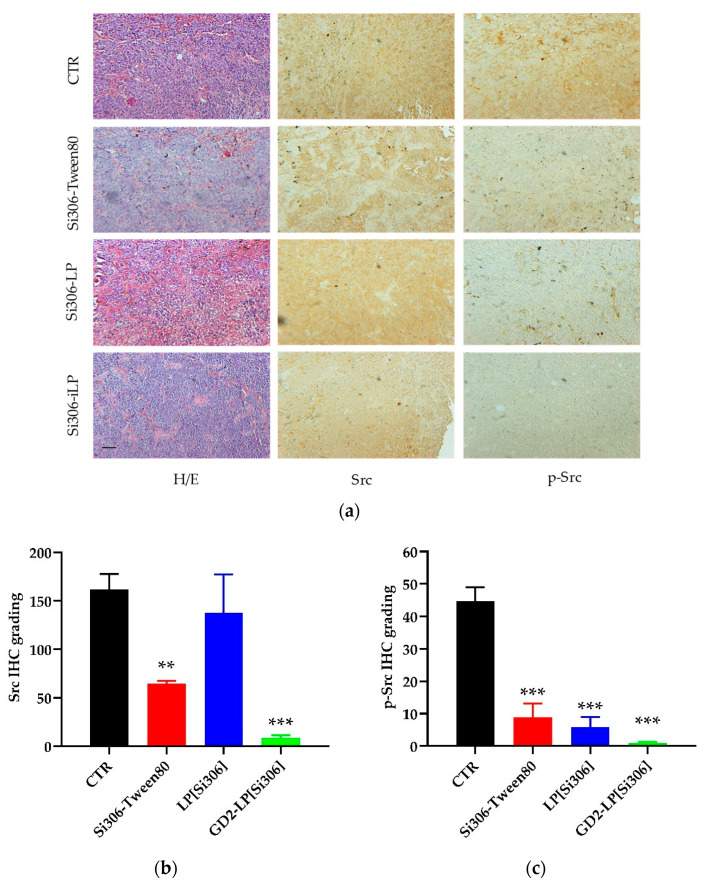

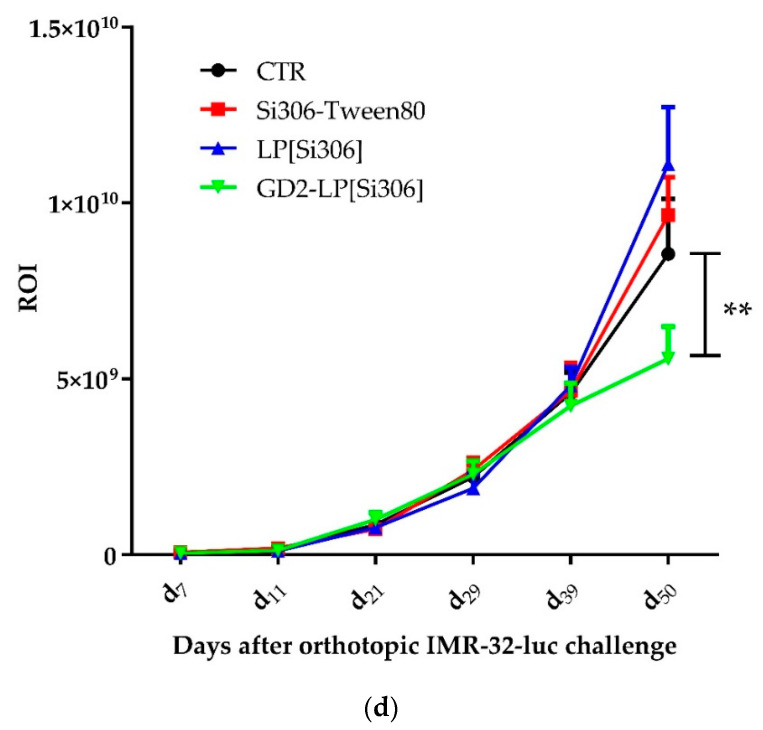
Si306 antitumor effect against an orthotopic animal model of neuroblastoma. Mice were injected in the adrenal gland with IMR-32-luc cells and treated with 5 mg/kg Si306, either free (Si306-Tween80) or encapsulated into untargeted and GD2-targeted liposomes (LP[Si306] and GD2-LP[Si306], respectively), as described in the Materials and Methods section. (**a**) Representative images of histology (H/E) analysis and IHC staining (brown) for Src and p-Src proteins from tumor tissues of untreated (CTR) or treated mice (more brown staining, more Src and p-Src proteins expression). Bar: 200 μm. (**b**,**c**) Digital analyses of IHC grading of tumor tissues from the different experimental conditions. Histogram results from analysis of acquired images after Src (**b**) and p-Src (**c**) staining. Columns: mean ± SD. ** *p* ˂ 0.01 and *** *p* ˂ 0.001: vs. CTR. (**d**) Tumor growth delay followed by BLI imaging. Photon counts in the tumor Region of Interest (ROI) are reported. ** *p* ˂ 0.01: GD2-LP[Si306] vs. CTR, Si306-Tween80 and LP[Si306].

In the first set of in vivo studies, IMR-32-luc-bearing mice were treated with 5 mg/kg of Si306, either dissolved in a mixture of Tween80 (10% *v*/*v*), encapsulated in untargeted (LP[Si306]) or GD2-targeted (GD2-LP[Si306]) liposomes, for eight times total. The growth of the tumor masses and the response to the therapy were monitored daily by bioluminescence imaging (BLI). During the experiment, five mice/group were euthanized 24 h after the fifth treatment, and tumor masses recovered and processed for histological (IHC) studies. Tumors demonstrated generally a tight dysplastic phenotype in all experimental points as shown in the representative images of H/E staining (Figure 7a). Then, in order to evaluate the expression of c-Src and its activation status, representing the target of the inhibitor of c-Src tyrosine kinase Si306, tumor specimens were processed for the detection of total c-Src protein and its phosphorylated form (*p*-Y416-Src). Interestingly, compared to CTR tumors, both proteins were significantly down-regulated after Si306 treatments, with a major reduction of c-Src and p-Src in tumors from mice treated with GD2-LP[Si306] (Figure 7b,c), indicating the specific action of our NB-targeted liposomal formulation. Finally, mice treated with GD2-LP[Si306] showed a significant (*p* < 0.01) delay in tumor growth, compared with CTR mice and those treated with free Si306, or with Si306 encapsulated in untargeted liposomes (Figure 7d).

**Figure 8:**
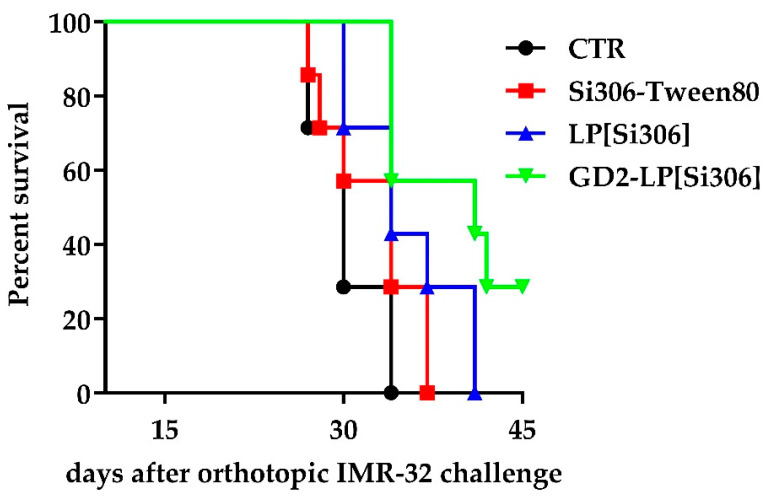
Survival curves of neuroblastoma-bearing mice treated with Si306. Mice were injected in the adrenal gland with IMR-32-luc cells and treated with 25 mg/kg Si306, either free (Si306-Tween80) or encapsulated into untargeted and GD2-targeted liposomes (LP[Si306] and GD2-LP[Si306], respectively), as described in the Materials and Methods section. Survival times of untreated (CTR) and treated mice.

In the second set of in vivo studies, the tumor response to treatment was evaluated by checking the animal survival. In this case, mice were treated with 25 mg/kg of Si306, following the schedule plan used for the imaging study. Mice treated with free Si306 did not show any increased life span compared to CTR (not statistically significant). On the contrary, compared to CTR mice, a significant increase of the survival time was observed in mice treated with Si306 encapsulated in untargeted (*p* = 0.0305) and, most of all, in GD2-targeted (*p* = 0.0029) liposomes (Figure 8 and Table 5), without signs of evident toxicity (such as apathy, hyperactivity, vomiting, diarrhea, morbidity, and weight lost. All together, these results indicate that our GD2-targeted formulation is long circulating, safe and able to exert a specific anti-NB effect also in vivo.

## 4. Discussion

In this study, we investigated the therapeutic potential of a c-Src tyrosine kinase inhibitor, the pyrazolo [3,4-*d*]pyrimidine derivative Si306, encapsulated in liposomal formulations specifically developed to recognize and kill neuroblastoma (NB) cells. The results obtained in this study utilizing both in vitro and in vivo models suggest a promising therapeutic profile of our strategy in high-risk NB.

c-Src belongs to the Src-family tyrosine kinases, which are involved in cancer development and invasiveness [62]. High levels of c-Src, as well as its aberrant activation, have been identified in several tumors [63], also including NB [64,65], thus representing an interesting therapeutic target. To date, different Src inhibitors have been developed [66]. In our laboratory, we have recently developed and characterized the compound Si306, which shows to have a favorable in vitro and in vivo profile of activity against NB models [22]. Nevertheless, the good antitumor activity of Si306 is associated with a sub-optimal aqueous solubility, which might hinder its further development. Here, we demonstrate that our liposomes encapsulating Si306, overcome its poor aqueous solubility, allowing to obtain suitable formulations for its in vivo use.

Size and ζ-potential are the most important features for the application of nanoparticles in preclinical and clinical setting. Particle size has, indeed, a significant impact on the circulation time, in particular affecting the rate of clearance by the mononuclear phagocyte system, where larger nanoparticles are faster recognized and eliminated [67]. Moreover, to facilitate the extravasation of nanoparticles from the blood stream into the tumor, the optimal size is around 80–150 nm [68]. ζ-potential, which indicates the degree of electrostatic repulsion between the particles, guarantees the stability of nanoparticles’ dispersion. Specifically, nanoparticles with ζ-potential values greater than +25 mV or less than −25 mV typically have high degree of stability [69].

In this study, Si306 was encapsulated in stealth liposomes, undecorated (LP[Si306]) or decorated (GD2-LP[Si306]) with a monoclonal antibody able to specifically recognize and bind to the disialoganglioside GD2 expressed by NB cells [36,48]. The disialoganglioside GD2 is highly expressed on NB cells [70] and recently, immunotherapy based on the use of anti-GD2 antibodies have been incorporated into standard of care treatment for patients with high-risk NB with clear benefit [39,40]. In our work, the GD2 receptor was instead used as an internalizing target ligand to allow nanotechnology-based Src inhibitors uptake from NB cells, leading to cytotoxic effects both in vitro and in vivo. Specifically, both untargeted and GD2-targeted liposomal formulations showed good morphological and physicochemical properties, which were maintained stable over a period of 14 days at storage conditions. These characteristics, together with an excellent drug encapsulation efficiency and its negligible leakage over a 96 h period, render our liposomes suitable for their in vitro and, most of all, in vivo use. Moreover, the achieved levels of anti-GD2 antibody coupled at the external surface of the liposomes, were estimated in a range that, in our experience, perfectly fits with the antibody/phospholipids ratio considered mandatory for nanoparticles to reach the preclinical setting.

When tested in vitro, Si306 shows its maximum antitumor activity when encapsulated inside GD2-targeted liposomes, resulting proportionally higher on the NB cells that more express the GD2 receptor on the cellular membrane. This result demonstrates that Si306 activity increases due to the active targeting of the nanoparticles to the target cells, as previously reported for other anticancer compounds [42,44,46,48].

Before moving to test the antitumor effectiveness of our liposomal formulations in a clinically relevant animal model of human NB [49], nanoparticles’ pharmacokinetic and tissue distribution profiles were evaluated in both healthy, immunocompetent, and tumor-bearing, immunodeficient, mice. In both cases, LP[Si306] and GD2-LP[Si306] formulations were compared to “free” Si306 dissolved in a solution containing Tween80 and benzyl alcohol as co-solvents [22]. The choice to administer the free Si306 inhibitor dissolved in this solution, rather than in DMSO [20] as for the in vitro tests, derives from previous observations that had highlighted a greater stability of the drug in this form, since it allowed the formation of micelle-like particles (unpublished data). Nevertheless, the delivery of Si306 by liposomes further increased its blood exposure in both mice strands. Si306-loaded, both untargeted and GD2-targeted, liposomal formulations have, indeed, good stability and long circulation times, becoming useful for subsequent therapeutic experiments in vivo. More interestingly, compared to both free Si306 and untargeted liposomes, GD2-LP[Si306] show a significant increase in tumor binding in vivo, confirming the cellular association results obtained in vitro, and enhance the life span of treated mice highlighting, once again, the importance of the antibody-driven targeting in the tumor recognition and in the Si306-derived antitumor efficacy.

As a note of caution, in the light of possible therapeutic developments in pre-clinical and in the clinic for NB, and also considering the numerous studies that have already investigated Src as antitumor target, we think that our data could be mainly useful for proposing new combination therapy, in which signaling pathways different from Src are also targeted. This strategy was successfully applied in different types of cancers [71,72]. Indeed, at present, only few Src inhibitors have been either approve by the FDA or are in ongoing clinical trials, and always in a combination therapy setting [73].

## 5. Conclusions

This study demonstrates that the efficient encapsulation of the inhibitor of the c-Src tyrosine kinase, Si306 into pegylated stealth liposomes decorated with anti-GD2 mAbs improves its anti-tumor effects, compared to Si306, either free or encapsulated into untargeted liposomes, against human neuroblastoma (NB) cells. The increased therapeutic efficacy was obtained thanks to the specific, GD2-mediated, NB cells recognition by GD2-targeted liposomes.

## Figures and Tables

**Table 1:** Properties of LP[Si306] and GD2-LP[Si306].

Parameters	LP[Si306]	GD2-LP[Si306]
Size (nm) ^a^	126 ± 15	133 ± 16
PDI ^a^	0.121 ± 0.015	0.120 ± 0.003
ζ-potentials (mV) ^a^	−22.6 ± 3.5	−19.3 ± 1.6
Thickness bilayer (nm) ^b^	7.02 ± 1.80	7.21 ± 1.10
Amount of PLs (μmol/mL) ^c^	9.06 ± 0.65	10.80 ± 1.84
EE% ^c^	76.91 ± 3.19	72.39 ± 3.75

^a^ measured by DLS (Dynamic Light Scattering); ^b^ measured by analysis of cryo-TEM images by Image J Software; ^c^ measured by UV/LC-MS. For all measurements the mean value ± S.D. is reported.

**Table 2:** Stability of LP[Si306] and GD2-LP[Si306] over a period of 14 days.

Parameters	Day 0	Day 3	Day 7	Day 14
	LP[Si306]			
Size (nm) ^a^	126 ± 15	126 ± 4	148.3 ± 11	152 ± 13
PDI ^a^	0.121 ± 0.015	0.122 ± 0.011	0.176 ± 0.032	0.300 ± 0.012
ζ-potential (mV) ^a^	−22.6 ± 3.5	−22.9 ± 3.1	−26.1 ± 3.2	−23.1 ± 2.6
	GD2-LP[Si306]			
Size (nm) ^a^	133 ± 16	134 ± 14	137 ± 12	144 ± 15
PDI ^a^	0.120 ± 0.003	0.121 ± 0.009	0.151 ± 0.012	0.212 ± 0.019
ζ-potential (mV) ^a^	−19.3 ± 1.6	−18.7 ± 1.3	−19.7 ± 1.7	−20.1 ± 1.5

^a^ Measured by DLS (dynamic light scattering).

**Table 3:** IC_50_ values of Si306 and liposomal formulations evaluated on NB cells.

Cell Lines	IC_50_ (µM) ± S.D. ^a^		
	Si306 in DMSO	LP[Si306]	GD2-LP[Si306]
IMR-32	5.9 ± 1.4	3.3 ± 0.5	2.3 ± 0.6
HTLA-230	2.9 ± 0.3	0.6 ± 0.1	0.5 ± 0.1
SH-SY5Y	19.6 ± 0.7	34.5 ± 1.3	16.8 ± 0.2

^a^ IC_50_ was evaluated by MTT assay. Data are means ± S.D. of three independent experiments each carried out in triplicate.

**Table 4:** Plasma PK parameters of Si306, either free (Si306-Tween80) or encapsulated into untargeted and GD2-targeted liposomes (LP[Si306] and GD2-LP[Si306], respectively) after i.v. administration of a single dose of 5 mg/kg in healthy mice.

Parameter ^a^	Unit	Plasma		
		Si306-Tween80	LP[Si306]	GD2-LP[Si306]
Dose	mg/kg	5	5	5
t_1/2_ ^b^	h	15.31	17.10	15.74
T_0_ ^c^	h	0.08	0.08	0.08
C_max_ ^d^	μg/mL	3.42	5.42	4.73
AUC_0__→48h_ ^e^	μg/mL × h	4.30	10.35	8.34
AUC_0__→__∞_ ^e^	μg/ML × h	4.59	11.73	9.05
MRT_0__→__∞_ ^f^	h	11.29	20.43	14.76
V_z_ ^g^	L/Kg	120.36	52.57	12.54
CL ^h^	L/h/Kg	5.45	2.13	0.55

^a^ Calculated with PKSolver; ^b^ t_1/2_: half-life. ^c^ T_0_: time of maximum concentration observed. ^d^ C_max_: maximum concentration observed. ^e^ AUC: area under the curve. ^f^ MRT: mean residence time ^g^ V: volume of distribution. ^h^ CL: clearance. PK data were evaluated using a non-compartment model.

**Table 5:** Median survival days after treatments and statistics between experimental groups.

Treated Group	Median Survival (Days)
CTR	30
Si306-Tween80	34
LP[Si306]	34
GD2-LP[Si306]	41
Statistics (*p*-Value ^a^)	
CTR vs. LP[Si306]	0.0305 (*)
CTR vs. GD2-LP[Si306]	0.0029 (**)
Si306-Tween80 vs. GD2-LP[Si306]	0.0191 (*)

^a^ Tukey’s two-way multiple comparisons test (ANOVA) was performed to test the significance of the observed differences (* *p* < 0.05 and ** *p* < 0.01).

## Data Availability

Not applicable.

## Supplementary Materials

The following are available online at https://www.mdpi.com/article/10.3390/biomedicines10030659/s1. Figure S1: Analysis of c-Src and p-Src proteins by western blotting in lysate from IMR-32, HTLA-230, and SH-SY5Y neuroblastoma cell lines. GAPDH protein: loading control; Figure S2: Biodistribution profiles in liver, spleen and kidneys from healthy mice of (a) Si306-Tween80, (b) LP[Si306] and (c) GD2-LP[Si306] at the dosage 5 mg/kg over 48 h (mean ± S.E.M., *n* = 5); Figure S3: Plasma concentration-time curves (mean ± S.E.M., *n* = 5) after i.v. administration, in IMR-32-luc-bearing mice, of a single dose of 5 mg/kg Si306, either free (Si306-Tween80) or encapsulated into untargeted and GD2-targeted liposomes (LP[Si306] and GD2-LP[Si306], respectively), The plasma concentration in the y-axis is expressed as log_10_ scale; Figure S4: Representative LC-MS/MS chromatogram of Si306 and Si34 (I.S.); Table S1: AUC_0→48h_ of liver, spleen, and kidneys after i.v. administration of Si306, formulated as Tween80 solution and both liposomal formulations at a single dose of 5 mg/kg in healthy mice; Table S2: PK parameters of a single dose of 5 mg/kg Si306, either free (Si306-Tween80) or encapsulated into untargeted and GD2-targeted liposomes (LP[Si306] and GD2-LP[Si306], respectively) after i.v. administration, in IMR-32-luc-bearing mice; Table S3: Matrix Effect and Recovery of Si306 in mouse plasma and liver, spleen and kidney tissues; Table S4: Process Efficiency of LC-MS/MS using ESI interface.
