# Supplementary material for: The Pyrazolo[3,4-d]Pyrimidine Derivative Si306 Encapsulated into Anti-GD2-Immunoliposomes as Therapeutic Treatment of Neuroblastoma
Source: Biomedicines. 2022 Mar 12;10(3):659. doi: 10.3390/biomedicines10030659 (PMC8945814; doi:10.3390/biomedicines10030659)
Supplement: Supplementary file 1 [file biomedicines-10-00659-s001.zip › biomedicines-1617649-supplementary.pdf]

biomedicines

MDPI

Supplementary Materials

The Pyrazolo[3,4-*d*]Pyrimidine Derivative Si306 Encapsulated Into Anti-GD2-Immunoliposomes as Therapeutic Treatment of Neuroblastoma

Enrico Rango [1,†], Fabio Pastorino [2,*,†], Chiara Brignole [2], Arianna Mancini [1], Federica Poggialini [1], Salvatore Di Maria [1], Claudio Zamperini [3], Giulia Iovenitti [1], Anna Lucia Fallacara [1], Samantha Sabetta [4], Letizia Clementi [4], Massimo Valoti [5], Silvia Schenone [6], Adriano Angelucci [4], Mirco Ponzoni [2,‡], Elena Dreassi, [1,*,‡] and Maurizio Botta [1,3,7,‡]

[1] Dipartimento Biotecnologie, Chimica e Farmacia, Università degli Studi di Siena, 53100 Siena, Italy; rango.enrico@gmail.com (E.R.); arianna.mancini3@gmail.com (A.M.); federicapoggialini91@gmail.com (F.P.); dimaria6@student.unisi.it (S.D.M.); iovenitti.giulia@gmail.com (G.I.); al.fallacara@gmail.com (A.L.F.); botta.maurizio@gmail.com (M.B.)
[2] Laboratory of Experimental Therapies in Oncology, IRCCS Istituto G. Gaslini, 16148 Genoa, Italy; chiarabrignole@gaslini.org (C.B.); mircoponzoni@gaslini.org (M.P.)
[3] Lead Discovery Siena S.r.l., Via Vittorio Alfieri 31, 53019 Castelnuovo Berardenga, Italy; claudiozamperini@gmail.com
[4] Dipartimento Scienze Cliniche Applicate e Biotecnologiche, Università dell'Aquila, Via Vetoio, 67100 Coppito, Italy; samantha.sabetta@graduate.univaq.it (S.S.); letizia.clementi@graduate.univaq.it (L.C.); adriano.angelucci@univaq.it (A.A.)
[5] Dipartimento Scienze della Vita, Università degli Studi di Siena, Via Aldo Moro 2, 53100 Siena, Italy; massimo.valoti@unisi.it
[6] Dipartimento di Farmacia, Università degli Studi di Genova, Viale Benedetto XV 3, Genoa 16132, Italy; schenone@difar.unige.it
[7] Sbarro Institute for Cancer Research and Molecular Medicine, Center for Biotechnology, College of Science and Technology Temple University, BioLife Science Building, Suite 333, 1900 North 12th Street, Philadelphia, PA 19122, USA
* Correspondence: fabiopastorino@gaslini.org (F.P.); elena.dreassi@unisi.it (E.D.); Tel.:+010-56363541 (F.P.); +0577-232039 (E.D.)
† Both authors contributed equally to this work.
‡ These authors share last authorship.

1. c-Src expression in IMR-32 and Fibroblasts cells

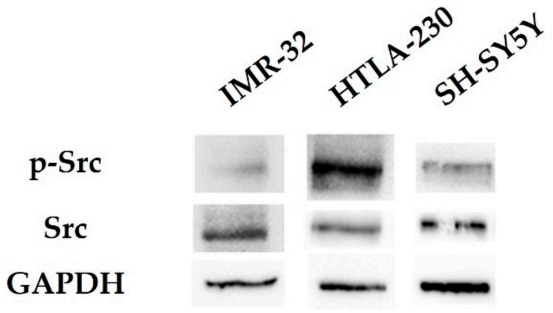

Figure S1. Analysis of c-Src and p-Src proteins by Western Blotting in lysate from IMR-32, HTLA-230 and SH-SY5Y neuroblastoma cell lines. GAPDH protein: loading control.

2. Biodistribution profiles over 48h of Si306-Tween80, LP[Si306] and GD2-LP[Si306] in healthy mice

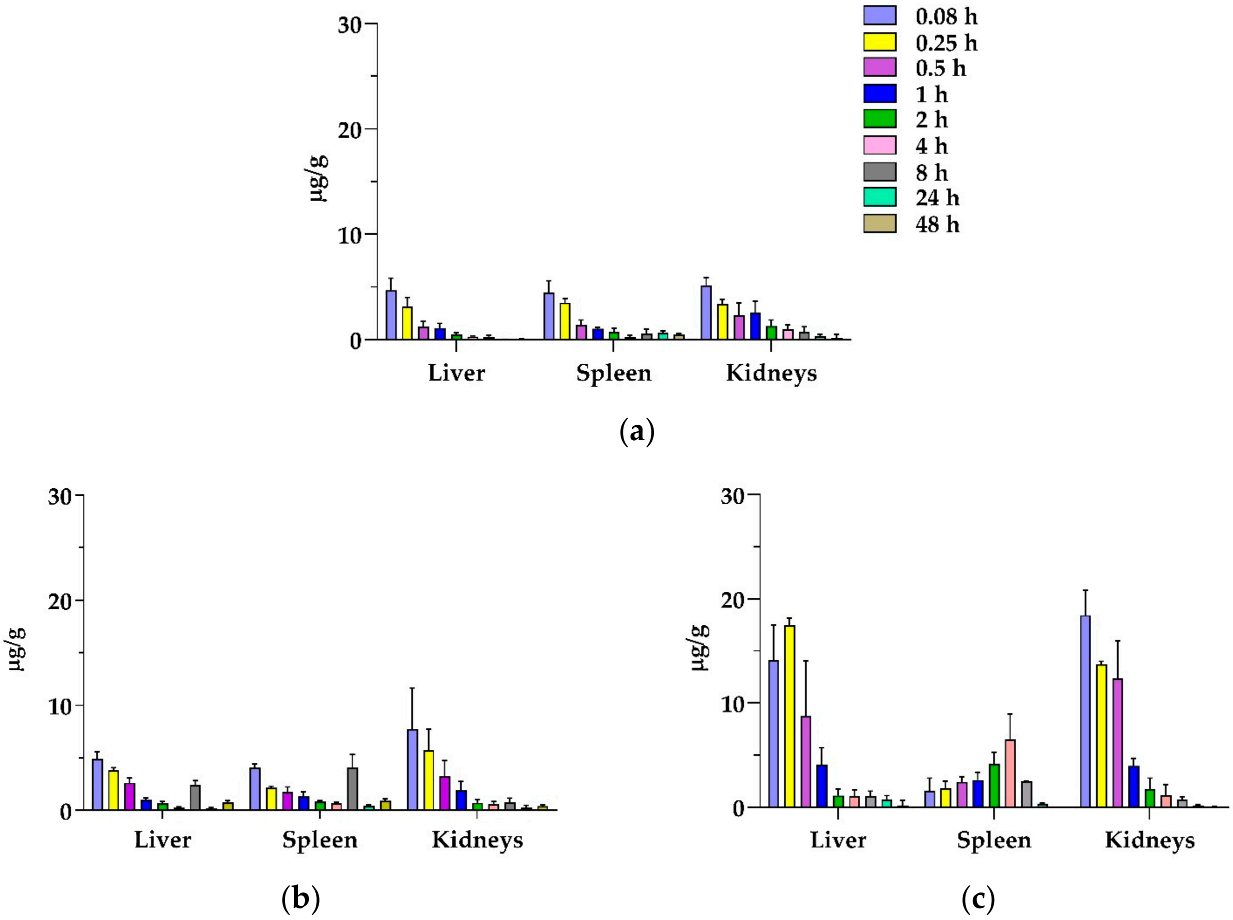

Figure S2. Biodistribution profiles in liver, spleen and kidneys from healthy mice of (**a**) Si306-Tween80, (**b**) LP[Si306] and (**c**) GD2-LP[Si306] at the dosage 5 mg/kg over 48 h (mean ± S.E.M., *n*=5).

Table S1. $AUC_{0\rightarrow48h}$ of liver, spleen, and kidneys after i.v. administration of Si306, formulated as Tween80 solution and both liposomal formulations at a single dose of 5 mg/kg in healthy mice.

	**$AUC_{0\rightarrow48h}$[a] (µg/mL×h)**		
**Tissue**	**Si306-Tween80**	**Si306-LP**	**Si306-iLP**
Liver	8.05	43.04	44.48
Spleen	29.28	67.04	60.43
Kidneys	24.60	26.43	31.01

[a]Area Under the Curve evaluated using a non-compartment model (PKSolver Software).

3. PK profile of Si306-Tween80, LP[Si306] and GD2-LP[Si306] in NB orthotopic murine model

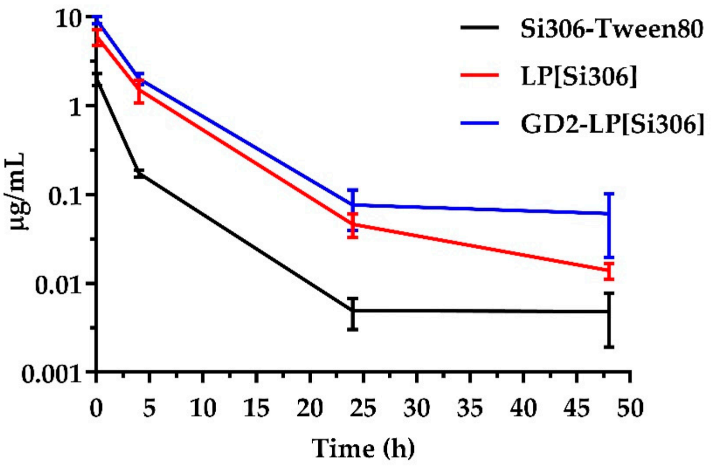

Figure S3. Plasma concentration-time curves (mean ± S.E.M., *n*=5) after i.v. administration, in IMR-32-luc-bearing mice, of a single dose of 5 mg/kg Si306, either free (Si306-Tween80) or encapsulated into untargeted and GD2-targeted liposomes (LP[Si306] and GD2-LP[Si306], respectively), The plasma concentration in the y-axis is expressed as log_{10} scale.

Table S2. PK parameters of a single dose of 5 mg/kg Si306, either free (Si306-Tween80) or encapsulated into untargeted and GD2-targeted liposomes (LP[Si306] and GD2-LP[Si306], respectively) after i.v. administration, in IMR-32-luc-bearing mice.

Parameter[a]	Unit	Plasma		
		**Si306-Tween80**	**Si306-LP**	**Si306-iLP**
Dose	mg/kg	5	5	5
$t_{1/2}$[b]	h	8.83	6.63	9.29
T_0[c]	h	0.08	0.08	0.08
C_{max}[d]	μg/mL	1.98	5.95	9.11
$AUC_{0\rightarrow 48h}$[e]	μg/mL×h	6.27	31.32	45.06
$AUC_{0\rightarrow\infty}$[e]	μg/mL×h	6.33	31.45	45.97
$MRT_{0\rightarrow\infty}$[f]	h	2.80	3.59	5.05
V_z[g]	L/Kg	10.06	1.52	1.45
CL[h]	L/h/Kg	0.79	0.57	0.11

[a]Calculated with PKSolver; [b]$t_{1/2}$: half-life. [c]T_0: time of maximum concentration observed. [d]C_{max}: maximum concentration observed. [e]AUC: area under the curve. [f]MRT: mean residence time [g]V: volume of distribution. [h]CL: clearance. PK data were evaluated using a non-compartment model.

3. Evaluation of matrix effect and recovery

The evaluation of the possible absence or presence of matrix effect %ME (ionization suppression or ionization enhancement) was evaluated by analyzing 3 sets of solutions: Si306 solutions present in the neat reconstitution solvent (LC mobile phase) were directly analyzed at prefixed concentrations. Mouse plasma/organ samples were first extracted and spiked after extraction with Si306 in the same solvent (mobile phase). Any additional variability of the peak areas for Si306 than those observed in set A would be indicative of an effect of sample matrix since Si306 at the same concentrations were spiked into plasma/organ extracts. Si306 was spiked before extraction into plasma/organ samples as in set B.

Each concentration point for each set of solutions (A, B and C) was prepared in triplicate. By using the mean peak areas obtained in Si306 neat solution (A), the corresponding mean peak areas for Si306 spiked after extraction into plasma/organ extracts (B), and mean peak areas for Si306 spiked before extraction (C), the %ME, recovery (%RE) and process efficiency (%PE) can be calculated as follow [1,2].

$$\%ME = \frac{B}{A} \times 100 \quad (1)$$

$$\%RE = \frac{C}{B} \times 100 \quad (2)$$

$$\%PE = \frac{(\%ME \times \%RE)}{100} \quad (3)$$

The matrix effect during validation of analytical methods in biological samples can be best examined by comparing the MS/MS response (peak areas) of Si306 at a given concentration spiked post-extraction into plasma or tissue extract (B, Eq. 1), with the MS/MS response (A, Eq. 1) of the same analyte in the "neat" mobile phase. The matrix effect values, evaluated using the ESI interface in plasma and tissue extracts and calculated according to Eq. 1, are summarized in Table S3. A value of 100% indicates that the response in the mobile phase and in the plasma/tissue extracts were the same and no absolute matrix effect was observed. A value of >100% indicates an ionization enhancement and a value of <100% indicates ionization suppression.

Besides, recovery (%RE) is determined using Eq. 2 as the ratio of mean peak areas of Si306 spiked before extraction (C) and mean peak areas of Si306 spiked after extraction into plasma/organ extracts (B). In this way, "true" recovery values that are not affected by the matrix has been obtained.

Table S3. Matrix Effect and Recovery of Si306 in mouse plasma and liver, spleen and kidney tissues.

µg/mL	Matrix Effect (%)				Recovery (%)			
of Si306	Plasma	Liver	Kidneys	Spleen	Plasma	Liver	Kidneys	Spleen
0.1	62.77	-	-	-	109.73	-	-	-
1	73.75	112.03	132.08	131.62	106.50	115.61	99.06	99.48
5	-	-	-	-	-	-	-	-
10	83.62	133.89	130.83	132.18	104.76	95.49	104.12	93.19
50	94.99	-	-	-	100.42	-	-	-
100	-	135.99	135.50	106.09	-	97.12	97.05	86.27

%ME data suggest a moderate ionization enhancement for Si306 in all tissues while a very small ionization suppression for Si306 in plasma samples was observed. Ultimately, %ME and %RE data suggest that the developed LC-MS/MS analysis method can be successfully applied to the PK and BD studies for the determination of Si306 in biological plasma and tissues. Furthermore, from the evaluation of %ME and %RE it is possible to calculate the process efficiency (Table S4) which expresses the trueness of the LC-MS/MS instrument applying Eq. 3.

Table S4. Process Efficiency of LC-MS/MS using ESI interface.

µg/mL of Si306	Process Efficiency (%)			
	**Plasma**	**Liver**	**Kidney**	**Spleen**
0.1	68.88	-	-	-
1	78.55	129.52	130.84	130.93
10	87.60	127.85	136.22	123.18
50	95.30	-	-	-
100	-	132.06	131.51	91.53

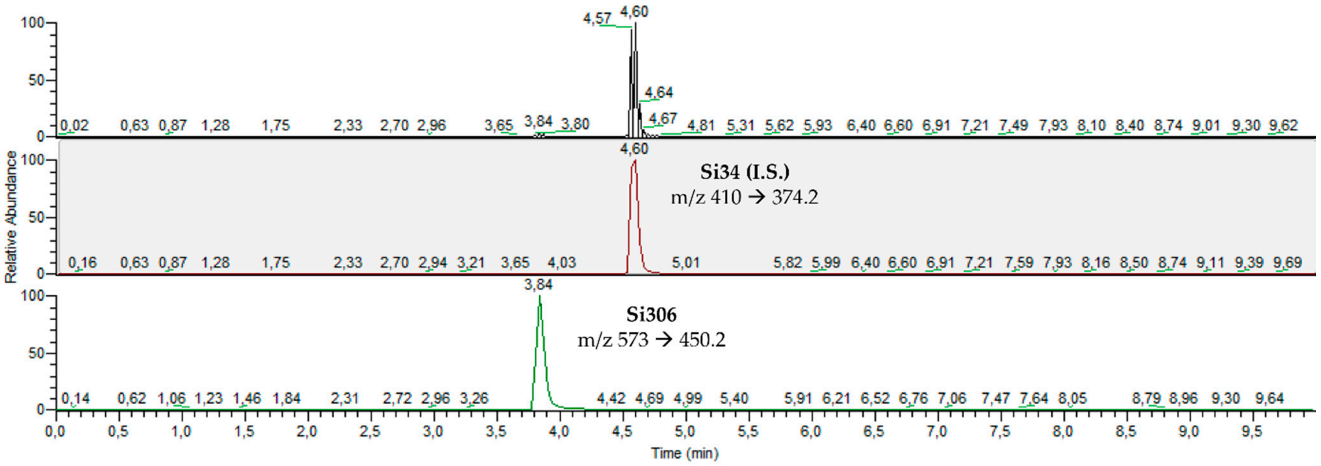

Figure S4. Representative LC-MS/MS chromatogram of Si306 and Si34 (I.S.).

References

1. Matuszewski, B.K.; Constanzer, M.L.; Chavez-Eng, C.M. Strategies for the assessment of matrix effect in quantitative bioanalytical methods based on HPLC-MS/MS. *Anal. Chem.* **2003**, *75*, 3019–3030. https://doi.org/10.1021/ac020361s.
2. Rango, E.; D'Antona, L.; Iovenitti, G.; Brai, A.; Mancini, A.; Zamperini, C.; Trivisani, C.I.; Marianelli, S.; Fallacara, A.L.; Molinari, A.; et al. Si113-prodrugs selectively activated by plasmin against hepatocellular and ovarian carcinoma. *Eur. J. Med. Chem.* **2021**, *223*, 113653. https://doi.org/10.1016/j.ejmech.2021.113653.
